# Modelling of Impulsional pH Variations Using ChemFET-Based Microdevices: Application to Hydrogen Peroxide Detection

**DOI:** 10.3390/s140203267

**Published:** 2014-02-19

**Authors:** Abdou Karim Diallo, Lyes Djeghlaf, Jerome Launay, Pierre Temple-Boyer

**Affiliations:** 1 CNRS, LAAS, 7 avenue du colonel Roche, F-31400 Toulouse, France; E-Mails: dialloabdoukarim@yahoo.fr (A.K.D.); ldjeghla@laas.fr (L.D.); jlaunay@laas.fr (J.L.); 2 University of Toulouse; UPS; LAAS; F-31400 Toulouse, France

**Keywords:** modelling, ChemFET, microelectrode, H_2_O electrolysis, H_2_O_2_ detection

## Abstract

This work presents the modelling of impulsional pH variations in microvolume related to water-based electrolysis and hydrogen peroxide electrochemical oxidation using an Electrochemical Field Effect Transistor (ElecFET) microdevice. This ElecFET device consists of a pH-Chemical FET (pH-ChemFET) with an integrated microelectrode around the dielectric gate area in order to trigger electrochemical reactions. Combining oxidation/reduction reactions on the microelectrode, water self-ionization and diffusion properties of associated chemical species, the model shows that the sensor response depends on the main influential parameters such as: (i) polarization parameters on the microelectrode, *i.e.*, voltage (V_p_) and time (t_p_); (ii) distance between the gate sensitive area and the microelectrode (d); and (iii) hydrogen peroxide concentration ([H_2_O_2_]). The model developed can predict the ElecFET response behaviour and creates new opportunities for H_2_O_2_-based enzymatic detection of biomolecules.

## 1. Introduction

Hydrogen peroxide (H_2_O_2_) is an important chemical species and its determination is of great interest for food processing, industrial, clinical and biochemical applications. In the past years, many methods for H_2_O_2_ detection were published in the literature. These different techniques involve spectrophotometry [1], chemiluminescence [2], titration [3] and fluorescence [4]. Nevertheless, they present considerable drawbacks, can be expensive and lack appropriate simplicity when used in specific applications. Electrochemistry is also a very interesting method for the detection of H_2_O_2_, leading to the development of associated sensors in liquid [5–8] and gaseous phases [9–11]. Indeed, amperometric or potentiometric electrochemical detection principles are advantageous over other methods due to their high sensitivity, fast response, low cost, simple instrumentation, compatibility with microtechnologies and related potentialities for miniaturization. The amperometric technique monitors redox phenomena in a conductive liquid/solid interface and has led to the development of electrochemical electrodes and cells [12,13] while the potentiometric one involves the detection of charges trapped at an insulating liquid/solid interface and has been responsible for the development of ion sensitive electrodes (ISE) and chemical field effect transistors (ChemFET) [13,14]. Both detection/transduction principles induced successful alternatives for the liquid phase analysis due to/in spite of different advantages/drawbacks in terms of (bio)chemical species detection, technological integration and data treatment. As a result, each of them was largely confined to specific applications according to their characteristics. Finally, the combination of amperometric and potentiometric techniques has also been very promising in terms of detection in liquid phase [15–19]. Such combination is associated with the functional integration of an electrochemical microelectrode and a pH-ChemFET on a single chip, leading to the realization of a pH-ChemFET based coulometric sensor-actuator system [15] also known as electrochemical field effect transistor (ElecFET) [19].

In parallel with the technological development of pH-ChemFET based microdevices, modelling investigations were also conducted. Based on the site-binding theory [20,21], such studies led to the development of complete behavioural models using SPICE [22], MATHEMATICA [23] or VHDL-AMS [24] softwares. From a general point of view, these different models focus on the study of electrolyte/insulator/semiconductor capacitive structures while taking into account the FET electrical behaviour due to well-known current-voltages equations [24,25]. Beyond the pH measurement, it is necessary to consider other phenomena occurring in liquid phase such as diffusion, migration, acid/basic chemical reactions, enzymatic biochemical reactions, …. This was performed for EnFET microsensors [26,27] as well as for pH-ChemFET-based coulometric sensor-actuator system [28,29]. Nevertheless, such modelling efforts have to be carried out in order to completely understand the ElecFET detection/transduction principles.

In this paper, we report modelling of ElecFET microdevices in the frame of water electrolysis and hydrogen peroxide oxidation using the MATLAB™ software, studying main influential parameters (polarization voltage and time as well as integration level) while dealing with impulsional pH variations in microvolume and H_2_O_2_ detection.

## 2. Experimental Section

The ElecFET microdevice was developed through functional integration of a metallic microelectrode with a pH-sensitive chemical field effect transistor (pH-ChemFET) on a silicon chip [19]. Thus, by combining at the microscale amperometric production of water-based ions in solution and pH potentiometric detection, unusual electrochemical detection properties were evidenced [15–19] and should be modelled accordingly.

**Figure 1.**
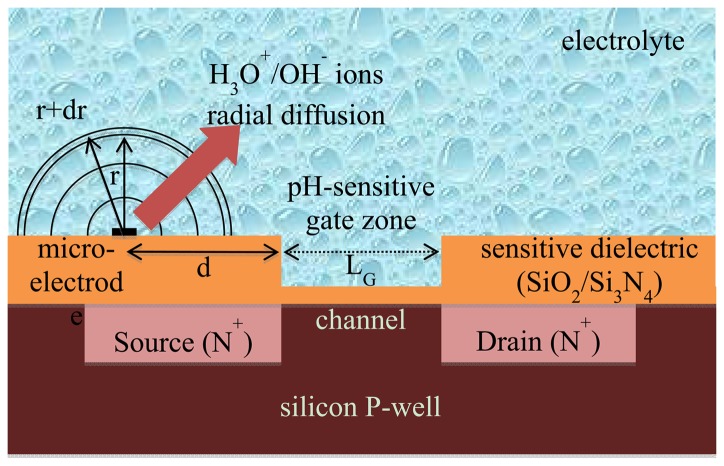
Cross-section of the ElecFET device describing the one-dimensional model in radial coordinate (scales are not respected for easiness of presentation).

Since the pH-ChemFET theoretical behaviour is well known [14,24,25], its modelling was considered globally by taking into account the threshold voltage variations with pH (see Section 2.3). Nevertheless, in order to completely understand the ElecFET detection/transduction principles, its technological processes and the associated planar structure should be described (Figure 1) [19,30]. At first, N-channel, field effect transistors (FET) were fabricated on silicon wafers using a standard P-well technology. Thus, heavily-doped, N^+^-type source (S) and drain (D) regions (thickness: ∼2 μm) were fabricated on a P-type silicon substrate to form the FET channel (length L_G_: ∼5 μm). Then, the pH-ChemFET gate was created. Typically, a 50 nm-thin silicon oxide SiO_2_ layer was thermally grown on the silicon substrate in order to obtain optimal gate dielectric properties and a 50 nm-thin Si_3_N_4_ layer was then deposited to provide pH detection properties. Finally, the metallic microelectrode was integrated on the device, next to the SiO_2_/Si_3_N_4_ pH-ChemFET sensitive gate (standard distances: 30–300 μm). Platinum was chosen since it is as an excellent catalyst for hydrogen peroxide oxidation [5,6,31]. Practically, platinum metallization (thickness: ∼200 nm) was used with a tantalum underlayer (thickness: ∼50 nm) to assure adhesion on Si_3_N_4_ surface. Overall, platinum microelectrode (area: ∼0.3 mm^2^) and pH-ChemFET (sensitive gate area: ∼4 × 10^−3^ mm^2^) were placed sufficiently close to one another in order to easily perform liquid phase analysis while keeping an adequate distance to avoid electrical interferences.

Concerning the operating principle, a typical electrical configuration was used [19]. On one hand, the pH-ChemFET electrical bias was achieved through a constant drain-source voltage V_DS_ and drain-source current I_DS_. Gate-source voltage V_GS_ was measured due to a reference electrode biasing the electrolyte to the mass (V_G_ = V_RE_ = V_electrolyte_ = 0). On the other hand, a suitable electrical polarization (voltage V_P_, time t_P_) was applied on the integrated platinum microelectrode and a standard platinum counter-electrode in the solution was used to complete the set-up (V_CE_ ≈ V_electrolyte_ = 0).

The ElecFET modelling takes into account the different chemical and physical phenomena occurring in the environment around the ElecFET microdevice: electrochemical production/consumption of water-based acid/basic species (hydronium H_3_O^+^ and hydroxide OH^−^ ions) due to redox phenomena on the integrated microelectrode, water self-ionization and diffusion phenomena in water medium. We assumed that (i) the transport by electromigration of H_3_O^+^ and OH^−^ ions is negligible and (ii) any similar phenomenon is induced by other ions in the electrolyte. Similarly, transport by convection was neglected. As a result, modelling procedure is described as follows:

- Study of the electrochemical production of water-based ions (H_3_O^+^ and OH^−^) on the integrated microelectrode,
- Study of the influence of water self-ionization,
- Study of diffusion phenomena in liquid phase, determination of water-based ion concentration profiles and pH distribution around the pH-ChemFET sensitive gate,
- Final determination of the pH-ChemFET potentiometric response (by considering only the typical “Nernst law” equation).

### 2.1. Modelling of the Electrochemical Production of Water-Based Ions

The ElecFET detection principles were first applied to water electrolysis phenomena triggered by an electrical polarization (voltage V_P_, time t_P_) on the integrated microelectrode:

- H_2_O oxidation (V_P_ > E_0+_): 6 H_2_O ----> O_2_ + 4 H_3_O^+^ + 4 e^−^
- H_2_O reduction (V_P_ < E_0−_): 4 H_2_O + 4e^−^ ----> 2 H_2_ + 4 OH^−^

where E_0+_ and E_0−_ are equilibrium potentials of oxidation and reduction reactions for water electrolysis respectively (E_0+_ ≈ 1.2 V and E_0−_ ≈ −0.8 V [32]).

The choice of a constant voltage to bias the integrated microelectrode was not compulsory in the frame of the ElecFET modelling: a constant current bias could have also been used. It was done in order to fit with ElecFET experimental characterisations previously performed [19]. Thus, according to laws of electrochemistry applied to a standard redox reaction (ox + ne^−^ ↔ red), the current I on the electrode is related to the electrode potential V through the following equation [32]:

(1) $$\text{I}=\text{nFS}\left\lfloor {\text{k}}_{\text{ox}}\left[\text{red}\right]\exp\left(\frac{\alpha \text{nF}}{\text{RT}}\text{V}\right)-{\text{k}}_{\text{red}}\left[\text{ox}\right]\exp\left(-\frac{\beta \text{nF}}{\text{RT}}\text{V}\right)\right\rfloor$$

where F is the Faraday constant (F = 96,485 C/mol), S is the electrode surface, k_ox_ and k_red_ are the standard rate constants of oxidation and reduction respectively, α and β are the anodic and cathodic transfert coefficients respectively (α + β = 1), R is the ideal gas constant (R = 8.32 J/(K·mol)) and T is the absolute temperature (K).

If the electrode potential V is higher than the oxidation potential E_+_, the reduction current can be neglected and Equation (1) becomes:

(2) $$\begin{array}{c} \text{I}={\text{nFSk}}_{\text{ox}}\left[\text{red}\right]\exp\left(\frac{\alpha \text{nF}}{\text{RT}}\text{V}\right)={\text{nFSk}}_{+}\left[\text{red}\right]\exp\left(\frac{\alpha \text{nF}}{\text{RT}}\left(\text{V}-{\text{E}}_{+}\right)\right)\\ {\text{k}}_{+}={\text{k}}_{\text{ox}}\exp\left(\frac{\alpha \text{nF}}{\text{RT}}{\text{E}}_{+}\right) \end{array}$$

Thus, in the case of monovalent ions, considering an elementary volume (surface S, thickness dr), the current I is given by:

(3) $$\text{I}=\text{eNSdr}\frac{\text{d}\left[\text{ox}\right]}{\text{dt}}$$

where N is the Avogadro number (N = 6.02 × 10^23^).

From Equations (2) and (3), temporal variations of the oxidant species concentration is finally given by:

(4) $$\frac{\text{d}\left[\text{ox}\right]}{\text{dt}}=\text{n}\frac{{\text{k}}_{+}}{\text{dr}}\left[\text{red}\right]\exp\left(\frac{\alpha \text{nF}}{\text{RT}}\left(\text{V}-{\text{E}}_{+}\right)\right)$$

In the same way, if the electrode potential V is lower than the reduction potential E_−_, the temporal variations of the reducer species concentration is given by:

(5) $$\begin{array}{c} \frac{\text{d}\left[\text{red}\right]}{\text{dt}}=\text{n}\frac{{\text{k}}_{-}}{\text{dr}}\left[\text{red}\right]\exp\left(\frac{\alpha \text{nF}}{\text{RT}}\left({\text{E}}_{-}-\text{V}\right)\right)\\ {\text{k}}_{-}={\text{k}}_{\text{red}}\exp\left(-\frac{\alpha \text{nF}}{\text{RT}}{\text{E}}_{-}\right) \end{array}$$

By applying Equations (4) and (5) to water oxidation and reduction respectively while considering that α = β = 0.5 [32], the temporal variations of H_3_O^+^ and OH^−^ ion concentrations at the microelectrode surface (r = 0, *cf.* Figure 1) are finally given by:

(6) $${\text{V}}_{\text{P}}>{\text{E}}_{0+}:{\left(\frac{\partial \left[{\text{H}}_{3}{\text{O}}^{+}\right]}{\partial \text{t}}\right)}_{\text{r}=0}={\text{G}}_{+}=4\frac{{\text{k}}_{0+}}{\text{dr}}\exp\left(\frac{2\text{F}}{\text{RT}}\left({\text{V}}_{\text{P}}-{\text{E}}_{0+}\right)\right)$$

(7) $${\text{V}}_{\text{P}}<{\text{E}}_{0-}:{\left(\frac{\partial \left[{\text{HO}}^{-}\right]}{\partial \text{t}}\right)}_{\text{r}=0}={\text{G}}_{-}=4\frac{{\text{k}}_{0-}}{\text{dr}}\exp\left(\frac{2\text{F}}{\text{RT}}\left({\text{E}}_{0-}-{\text{V}}_{\text{P}}\right)\right)$$

where k_0+_ and k_0−_ are standard rate constants of oxidation and reduction respectively (estimated respectively at 3 × 10^−9^ m/s and 10^−7^ m/s using the Tafel experimental method [32], results not shown), F is the Faraday constant (F = 96,485 C/mol), R is the ideal gas constant (R = 8.32 J/(K·mol)) and T is the absolute temperature (K).

In presence of hydrogen peroxide in solution (concentration [H_2_O_2_]), the associated oxidation reaction should also be taken into account on the integrated microelectrode:

H_2_O_2_ oxidation (V_P_ > E_1+_): H_2_O_2_ + 2 H_2_O → O_2_ + 2 H_3_O^+^ + 2 e

where E_1+_ is the equilibrium potential for the H_2_O_2_ oxidation (E_1+_ ≈ 0.3 V for pH = 7 [32]).

**Figure 2.**
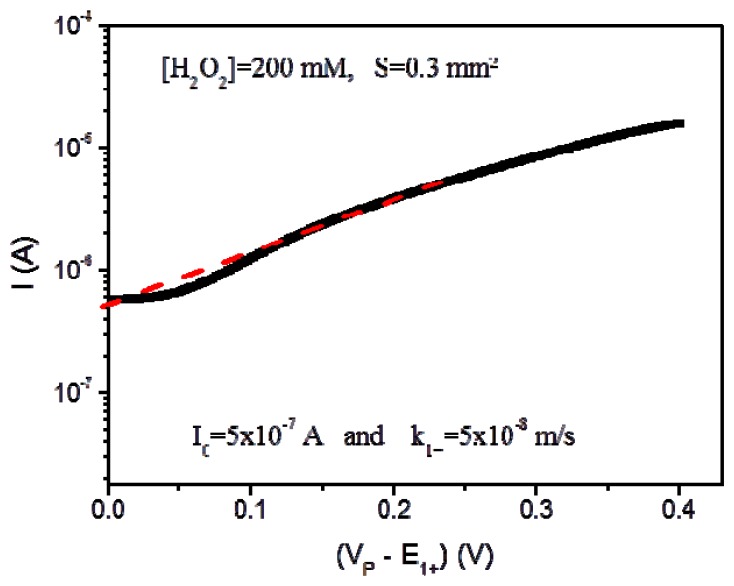
Definition of the standard rate constant k_1+_ for the H_2_O_2_ oxidation using the Tafel method (S = 0.3 mm^2^, [H_2_O_2_] = 200 mM).

As previously stated, according to Equation (4), temporal variations of [H_3_O^+^] concentration at the microelectrode surface (r = 0, *cf.* Figure 1) are given by:

(8) $$-{\text{V}}_{\text{P}}>{\text{E}}_{1+}:{\left(\frac{\partial \left[{\text{H}}_{3}{\text{O}}^{+}\right]}{\partial \text{t}}\right)}_{\text{r}=0}={\text{G}}_{1+}=2\left[{\text{H}}_{2}{\text{O}}_{2}\right]\frac{{\text{k}}_{1+}}{\text{dr}}\exp\left(\frac{\text{F}}{\text{RT}}\left({\text{V}}_{\text{P}}-{\text{E}}_{1+}\right)\right)$$

where k_1+_ is the standard rate constant for the H_2_O_2_ oxidation (estimated experimentally at 5 × 10^−8^ m/s from the current I_0_ obtained for V_P_ = E_1+_ in agreement with the Tafel method [32], Figure 2), F is the Faraday constant (F = 96,485 C/mol), R is the ideal gas constant (R = 8.32 J/(K·mol)) and T is the absolute temperature (K).

In our model, these equations were finally used to determine the electrochemical production of hydronium or hydroxide ions on the integrated microelectrode and its impact on the associated concentrations [H_3_O^+^] and [OH^−^].

### 2.2. Influence of Water Self-Ionization

In parallel, since the electrical polarization on the integrated microelectrode is responsible for the mass production of H_3_O^+^ or OH^−^ ion, water is locally set out of equilibrium from a chemical point of view. According to water self-ionization (2 H_2_O ↔ H_3_O^+^ + OH^−^), the kinetics of the [H_3_O^+^] and [OH^−^] concentrations are identical:

(9) $$\frac{\text{d}\left[{\text{H}}_{3}{\text{O}}^{+}\right]}{\text{dt}}=\frac{\text{d}\left[{\text{OH}}^{-}\right]}{\text{dt}}$$

Introducing a constant parameter c that depends of the initial difference between the [H_3_O^+^] and [OH^−^] concentrations, the mathematical integration of Equation (9) gives:

(10) $$\left[{\text{H}}_{3}{\text{O}}^{+}\right]=\left[{\text{OH}}^{-}\right]+\text{c}\Leftrightarrow \text{c}=\left[{\text{H}}_{3}{\text{O}}^{+}\right]-\left[{\text{OH}}^{-}\right]$$

Thus, considering that the return to equilibrium is associated with a steady-state regime [29], the final concentrations of hydronium and hydroxide ions [H_3_O^+^]_f_ and [OH^−^]_f_ are characterized by the following system (where K_w_ is the ionic product of water H_2_O):

(11) $$\{\begin{array}{l} {\left[{\text{H}}_{3}{\text{O}}^{+}\right]}_{\text{f}}{\left[{\text{OH}}^{-}\right]}_{\text{f}}={\text{K}}_{\text{W}}\\ {\left[{\text{H}}_{3}{\text{O}}^{+}\right]}_{\text{f}}={\left[{\text{OH}}^{-}\right]}_{\text{f}}+\text{c}\Leftrightarrow \text{c}={\left[{\text{H}}_{3}{\text{O}}^{+}\right]}_{\text{f}}-{\left[{\text{OH}}^{-}\right]}_{\text{f}} \end{array}$$

Solving this equation system yields:

(12) $${\left[{\text{H}}_{3}{\text{O}}^{+}\right]}_{\text{f}}^{2}-\text{c}{\left[{\text{H}}_{3}{\text{O}}^{+}\right]}_{\text{f}}-{\text{K}}_{\text{W}}=0$$

Considering the positive root of Equation (12), the [H_3_O^+^]_f_ and [OH^−^]_f_ values are finally given by:

(13) $${\left[{\text{H}}_{3}{\text{O}}^{+}\right]}_{\text{f}}=\frac{\text{c}+\sqrt{{\text{c}}^{2}+4{\text{K}}_{\text{W}}}}{2}$$

(14) $${\left[{\text{OH}}^{-}\right]}_{\text{f}}=\frac{\sqrt{{\text{c}}^{2}+4{\text{K}}_{\text{W}}}-\text{c}}{2}$$

Finally, the global variations of the [H_3_O^+^] and [OH^−^] concentrations related to the water self-ionization reaction are expressed by:

(15) $$\Delta \left[{\text{H}}_{3}{\text{O}}^{+}\right]=\Delta \left[{\text{OH}}^{-}\right]=\frac{\sqrt{{\left(\left[{\text{H}}_{3}{\text{O}}^{+}\right]-\left[{\text{OH}}^{-}\right]\right)}^{2}+4{\text{K}}_{\text{w}}}-\left[{\text{H}}_{3}{\text{O}}^{+}\right]-\left[{\text{OH}}^{-}\right]}{2}$$

Thus, in the developed model, any variations of [H_3_O^+^] and [OH^−^] concentrations were counterbalanced according to Equation (10) in order to take into account water self-ionization phenomena in solution.

It should be mentioned that the water self-ionization modelling was considered only in the case of pure water and therefore without considering any buffer properties. Such assumption is required to understand ElecFET detection/transduction principles. In practice, each solution of interest will have to be separately studied. Nevertheless, as demonstrated for urea-EnFET in the frame of haemodialysis [26,27,33], buffer effects will influence the ElecFET detection properties but will not drastically limit its operating principle.

### 2.3. Modelling of Diffusion Phenomena in Watery Phase

The diffusion model was adapted from a previous one developed for the EnFET-based microsensors and based on a finite element model implemented in MATLAB™ software [26,27]. It is associated with Fick's diffusion equation, assuming a one-dimensional model in spherical coordinates (Figure 1). The variable parameter is therefore the radius of the sphere r:

(16) $$\frac{\partial \text{C}\left(\text{r},\text{t}\right)}{\partial \text{t}}=\text{D}\frac{1}{{\text{r}}^{2}}\frac{\partial }{\partial \text{r}}\left({\text{r}}^{2}\frac{\partial \text{C}\left(\text{r},\text{t}\right)}{\partial \text{r}}\right)+\text{G}$$

where C(r,t) describes the concentration of H_3_O^+^ or OH^−^ ion for the studied case, D is the associated diffusion coefficient and G represents the chemical species generation according to the associated electrochemical reaction.

As a result, for the different studied cases, Equation (11) gives:

(17) $${\text{H}}_{2}\text{O}\;\text{oxidation}\;\left({\text{V}}_{\text{P}}>{\text{E}}_{0+}\right):\frac{\partial \left[{\text{H}}_{3}{\text{O}}^{+}\right]\left(\text{r},\text{t}\right)}{\partial \text{t}}={\text{D}}_{{\text{H}}_{3}{\text{O}}^{+}}\frac{1}{{\text{r}}^{2}}\frac{\partial }{\partial \text{r}}\left({\text{r}}^{2}\frac{\partial \left[{\text{H}}_{3}{\text{O}}^{+}\right]\left(\text{r},\text{t}\right)}{\partial \text{r}}\right)+{\text{G}}_{+}$$

(18) $${\text{H}}_{2}\text{O}\;\text{reduction}\;\left({\text{V}}_{\text{P}}<{\text{E}}_{0-}\right):\frac{\partial \left[{\text{OH}}^{-}\right]\left(\text{r},\text{t}\right)}{\partial \text{t}}={\text{D}}_{{\text{OH}}^{-}}\frac{1}{{\text{r}}^{2}}\frac{\partial }{\partial \text{r}}\left({\text{r}}^{2}\frac{\partial \left[{\text{OH}}^{-}\right]\left(\text{r},\text{t}\right)}{\partial \text{r}}\right)+{\text{G}}_{-}$$

(19) $${\text{H}}_{2}{\text{O}}_{2}\;\text{oxidation}\;\left({\text{V}}_{\text{P}}>{\text{E}}_{1+}\right):\frac{\partial \left[{\text{H}}_{3}{\text{O}}^{+}\right]\left(\text{r},\text{t}\right)}{\partial \text{t}}={\text{D}}_{{\text{H}}_{3}{\text{O}}^{+}}\frac{1}{{\text{r}}^{2}}\frac{\partial }{\partial \text{r}}\left({\text{r}}^{2}\frac{\partial \left[{\text{H}}_{3}{\text{O}}^{+}\right]\left(\text{r},\text{t}\right)}{\partial \text{r}}\right)+{\text{G}}_{1+}$$

In order to solve diffusion equations, the following initial and boundaries conditions were chosen:

(20) $$\{\begin{array}{l} \left[{\text{H}}_{3}{\text{O}}^{+}\right]\left(\text{r},0\right)=10^{-{\text{pH}}_{0}}\Leftrightarrow \left[{\text{OH}}^{-}\right]\left(\text{r},0\right)={\text{K}}_{\text{w}}10^{+{\text{pH}}_{0}}\\ {\left(\frac{\partial \text{C}\left(\text{r},\text{t}\right)}{\partial \text{t}}\right)}_{\text{r}=0}={\left(\frac{\partial \text{C}\left(\text{r},\text{t}\right)}{\partial \text{t}}\right)}_{\text{r}={\text{r}}_{\max}}=0 \end{array}$$

Thus, initial conditions assume spatially uniform concentrations of water-based ions and boundary conditions assume that no flux goes through the physical barrier of the sensor surface (r = 0) or far from it (r = r_max_ = 3 cm). By solving the mass transport equations system (Equations (17), (18) and/or (19)) using MATLAB™ software, the different ion concentration distributions [H_3_O^+^](r,t) and [OH^−^](r,t) were defined and the pH(r,t) function was deduced consequently.

### 2.4. Modelling of the pH-ChemFET Electrical Behaviour

Finally, concerning the pH-ChemFET electrical behaviour, modelling of the drain-source current I_DS_ as a function of gate-source and drain-source voltages V_GS_ and V_DS_, pH and any other interfering parameters was not performed. Indeed, since the pH-ChemFET device is well known at a theoretical level and was thoroughly studied in previous works [14,24,25], it was decided to take only into account its global detection properties associated with the Nernst's law. Thus, since the channel length L_G_ (typical values around 5 km [30]) is shorter than the distance d between the integrated microelectrode and the pH-ChemFET gate sensitive area (Figure 1), the pH-ChemFET threshold voltage V_T_ variation was estimated in a very simple way according to the following equation:

(21) $$\delta {\text{V}}_{\text{T}}\left(\text{t}\right)={\text{s}}_{0}\left(\text{pH}\left(\text{d},\text{t}\right)-{\text{pH}}_{0}\right)$$

where s_0_ is the pH-ChemFET sensitivity and d is the distance between the integrated microelectrode and the pH-ChemFET gate sensitive area (Figure 1).

## 3. Results and Discussion

Hereinafter, the ElecFET model is studied using the following parameters:

- T = 300 K and s_0_ = 60 mV/pH
- K_W_ = 10^−14^ (mol/L)^2^ and pH_0_ = 7
- D_H3O+_ = 9.3 × 10^−5^ cm^2^/s and D_OH−_ = 5.3 × 10^−5^ cm^2^/s
- S = 0.3 mm^2^ and d = 30 μm (if not indicated)

### 3.1. Modelling of Water Electrolysis Phenomena

**Figure 3.**
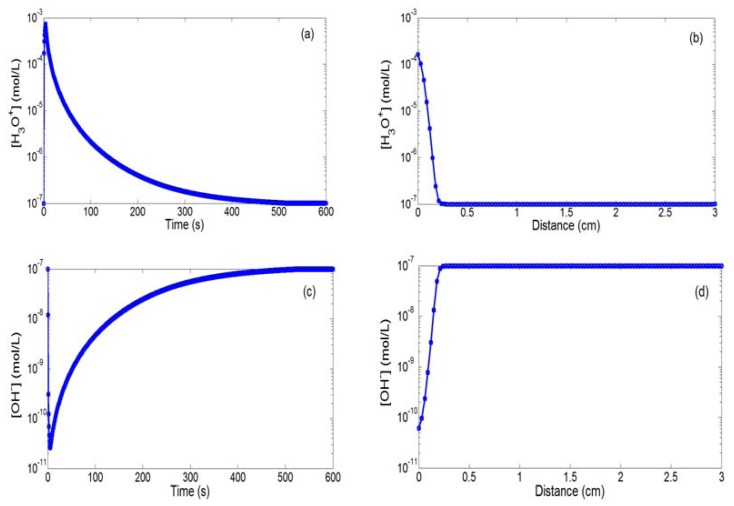
(**a,c**) Temporal (r = d = 30 μm) and (**b,d**) spatial variations (t = 12 s) of the [H_3_O^+^] and [OH^−^] concentrations for a positive bias on the integrated microelectrode (V_P_ = 1.23 V and t_P_ = 5 s).

The first study concerned modelling of a positive bias on the integrated microelectrode (polarization voltage V_P_ = 1.23 V and polarization time t_P_ = 5 s). In this case, water oxidation occurs on the integrated microelectrode, triggering the local production of H_3_O^+^ ions since V_P_ is higher than the water oxidation equilibrium potential E_0+_. Figure 3 show the temporal and spatial variations of hydronium (H_3_O^+^) and hydroxide (OH^−^) ion concentrations. As expected, the [H_3_O^+^] concentration (respectively, the [OH^−^] concentration) increases (respectively, decreases) very quickly by more than four decades (from 10^−7^ mol/L to 7 × 10^−4^ mol/L) and gradually takes its initial value (10^−7^ mol/L) as soon as the electrical bias on the integrated microelectrode is turned off (Figure 3a,c). Furthermore, it is obvious that these variations occur at millimetric distance (Figure 3b,d). This demonstrates that localized impulsional pH variations can be effectively obtained near the integrated microelectrode surface and can be detected at the adjacent pH-sensitive surface.

**Figure 4.**
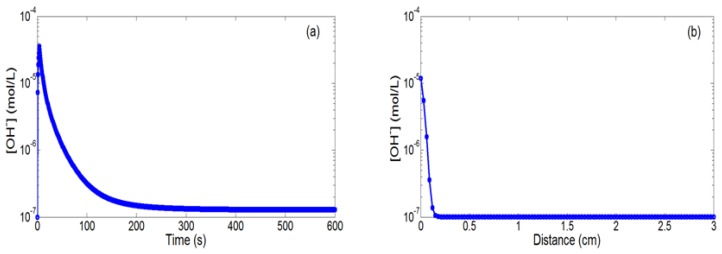
(**a**) Temporal (r = d = 30 μm) and (**b**) spatial variations (t = 12 s) of the [OH^−^] concentration for a negative bias on the integrated microelectrode (V_P_ = −0.85 V and t_P_ = 5 s).

In the case of negative bias (polarization voltage V_P_ = −0.85 V and polarization time t_P_ = 5 s), water reduction is taking place on the integrated microelectrode, triggering the local production of OH^−^ ions since the V_P_ is lower than the water reduction equilibrium potential E_0−_. Figure 4 illustrate respectively the temporal and spatial evolutions of the OH^−^ ion concentration. An [OH^−^] increase (from 10^−7^ mol/L to 4 × 10^−5^ mol/L) is noticed, followed by a return to equilibrium (10^−7^ mol/L) when the electrical bias is turned off. Of course, the H_3_O^+^ ion concentration follows opposite variations in agreement with the water acid/basic equilibrium (figures not shown). Impulsional pH variations are therefore evidenced at the millimetric scale, confirming finally that varied localized phenomena can be effectively obtained according to the polarization sign.

**Figure 5.**
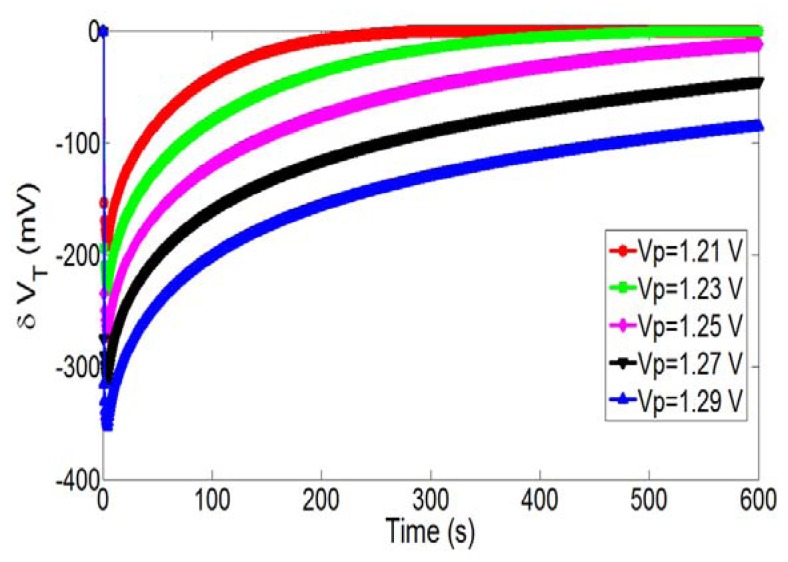
Temporal variations of the pH-ChemFET threshold voltage for different polarization voltages on the integrated microelectrode (V_P_ = 1.21, 1.23, 1.25, 1.27 and 1.29 V) and a given polarization time (t_P_ = 5 s).

Subsequently, we have studied the influences of the main parameters by focusing on the pH-ChemFET threshold voltage variations δV_T_. Figure 5 shows the associated temporal variations for different polarization voltages applied on the integrated microelectrode (V_P_ = 1.21, 1.23, 1.25, 1.27 and 1.29 V) and a given polarization time t_P_ = 5 s.

As expected, the polarization voltage increase is responsible for a local pH decrease and consequently a pH-ChemFET threshold voltage decrease. This behaviour corroborates the Butler-Volmer theory adapted to water oxidation (Equation (1)). Finally, since H_3_O^+^ ion production kinetics is an exponential function of V_P_, an increase in polarization voltage induces a quasi-linear decrease of the minimum voltage.

**Figure 6.**
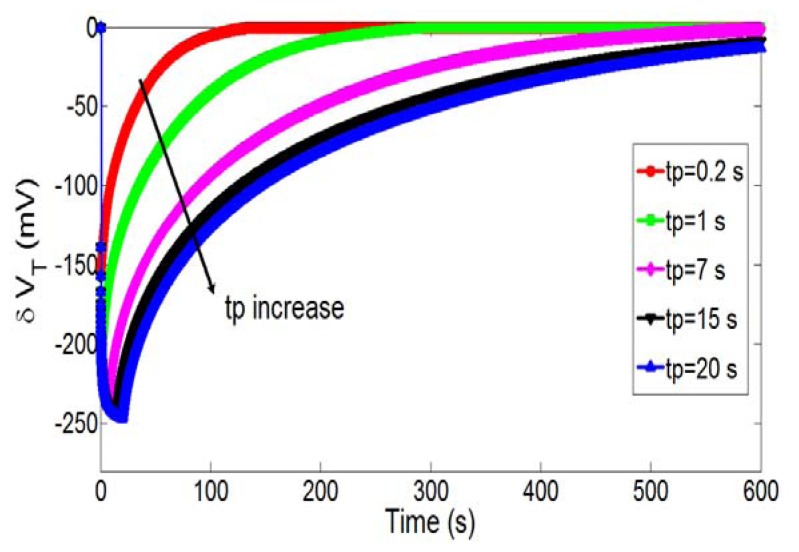
Temporal variations of the pH-ChemFET threshold voltage for a given polarization voltage on the integrated microelectrode (V_P_ = 1.23 V) and different polarization times (t_P_ = 0.2, 1, 7, 15 and 20 s).

Then, the influence of polarization time t_P_ was studied. Figure 6 illustrates the temporal variations of pH and associated pH-ChemFET threshold voltage for different polarization times (t_P_ = 0.2, 1, 7, 15 and 20 s) while keeping constant the polarization voltage on the integrated microelectrode (V_P_ = 1.23 V). In agreement with the electrochemical theory (Equation (1)), the polarization time increase is responsible for a local pH decrease and therefore a pH-ChemFET threshold voltage decrease. Nevertheless, pH variations are lower and tend to reach saturation.

**Figure 7.**
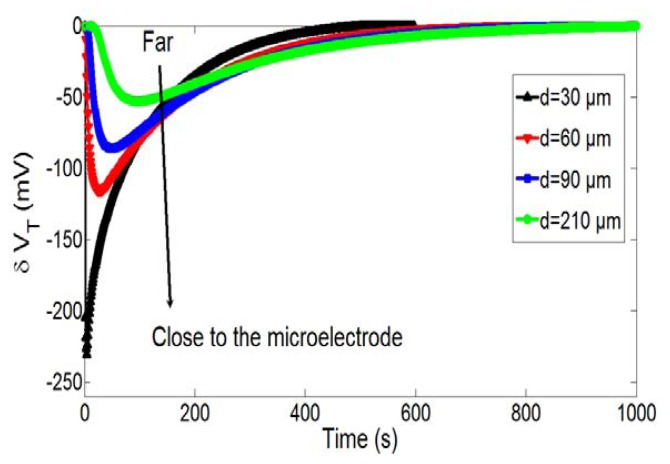
Temporal variations of the pH-ChemFET threshold voltage for different distances between the integrated microelectrode and the pH-sensitive gate (d = 30, 90, 150 and 210 μm) and a given polarization step (V_P_ = 1.23 V and t_P_ = 5 s).

Since diffusion phenomena control the ElecFET detection principles, spatial configuration should influence detection properties. To demonstrate such assumption, Figure 7 shows temporal variations of pH-ChemFET threshold voltage for different distances between the integrated microelectrode and the pH-ChemFET gate sensitive area (d = 30, 90, 150 and 210 μm) and for a given microelectrode polarization (V_P_ = 1.23 V, t_P_ = 5 s). It appears that the distance diminution is responsible for a local pH and therefore a pH-ChemFET threshold voltage decrease. This demonstrates that the ElecFET detection principles are effectively dependent on the spatial integration of both microelectrode and pH-ChemFET microdevices. For the highest integration levels (d ≪ 50 μm), very important localized impulsional pH variations are obtained for a given microelectrode polarization step (V_P_, t_P_). On the contrary, for the lowest integration levels (d ≫ 200 μm), phenomena are strongly attenuated, leading to low temporal pH wave and, consequently low ElecFET response. Since such pH variations were found to occur at millimetric distance (*cf.* Figure 3b,d), ElecFET detection potentialities is no longer possible when d parameter is higher than 1,000 μm, *i.e.*, when the functional integration between the microelectrode and the pH-ChemFET device is ineffective.

### 3.2. Modelling of Hydrogen Peroxide Oxidation Phenomena

**Figure 8.**
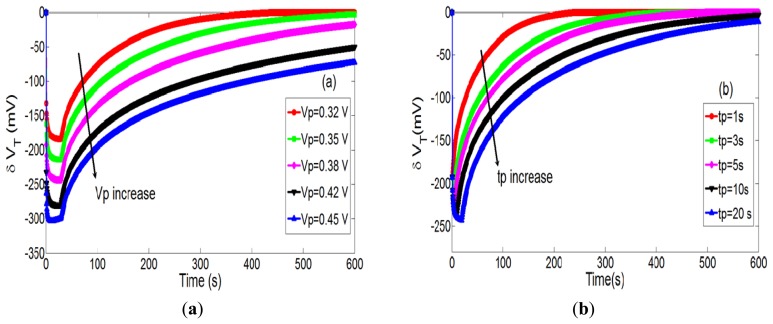
Temporal variations of the pH-ChemFET threshold voltage for different polarization steps, (**a**) V_P_ = 0.32, 0.35, 0.38, 0.42 and 0.45 V, (**b**) t_P_ = 1, 3, 5, 10 and 20 s, and for a given H_2_O_2_ concentration ([H_2_O_2_] = 45 mM).

Since hydrogen peroxide detection is of great interest and H_2_O_2_ was successfully studied using an ElecFET-based microdevice [17,19], our model was studied by taking into account the H_2_O_2_ oxidation on the integrated microelectrode. In this case, according to the hydrogen peroxide oxidation reaction, a positive bias on the integrated microelectrode is responsible for the electrochemical production of H_3_O^+^ ions and therefore a local pH decrease in solution (*cf.* Section 2.1). Since H_2_O and H_2_O_2_ molecules have similar electrochemical behaviours, impulsional pH variations localized at the microscale (typical dimension less than 1 mm) were also obtained in H_2_O_2_-rich solutions as soon as an appropriate polarization step (V_P_, t_P_) was applied (result not shown). In this case, phenomena occur at lower voltage according to the associated equilibrium potential E_1+_ value (E_1+_ ≈ 0.3 V for pH = 7 [32]). This is clearly evidenced on Figure 8 illustrating temporal variations of pH-ChemFET threshold voltage for different polarization voltages (V_P_ = 0.32, 0.35, 0.38, 0.42 and 0.45 V) and different polarization times (t_P_ = 1, 3, 5, 10 and 20 s), considering an H_2_O_2_ concentration of 45 mM. In this case, local impulsional pH variations are not so obvious. Indeed, during the polarization duration (t_P_ = 30 s), hydrogen peroxide oxidation is limited by the H_2_O_2_ diffusion phenomena towards the microelectrode surface. As a result, pH variations are no longer impulsional and higher polarization durations are required to reach a steady-state regime depending on the polarization voltage V_P_ (as well as on the [H_2_O_2_] concentration, see hereafter). Then, as previously stated, since the H_2_O_2_ oxidation reaction is controlled by the Butler-Volmer theory (Equation (3)), the polarization voltage V_P_ increase is responsible for a quasi-linear threshold voltage decrease while the polarization time t_P_ increase is associated to saturation phenomena.

**Figure 9.**
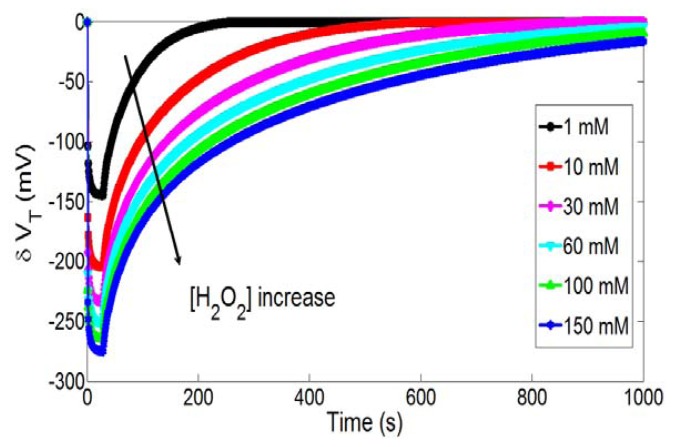
Temporal variations of the pH-ChemFET threshold voltage for different H_2_O_2_ concentrations ([H_2_O_2_] = 1, 10, 30, 60, 100 and 150 mM) and a given polarization step (V_P_ = 0.35 V and t_P_ = 30 s).

Finally, in the case of hydrogen peroxide detection, the Butler-Volmer theory emphasizes on the influence of the [H_2_O_2_] concentration on H_3_O^+^ ion production kinetics (Equation (3)). In order to study this influence, Figure 9 shows temporal variations of the pH-ChemFET threshold voltage for different H_2_O_2_ concentrations ([H_2_O_2_] = 1, 10, 30, 60, 100 and 150 mM) and a given polarization step (V_P_ = 0.35 V and t_P_ = 30 s). As expected, it is obvious that the hydrogen peroxide concentration increase is responsible for a local pH decrease and therefore a pH-ChemFET threshold voltage decrease.

**Figure 10.**
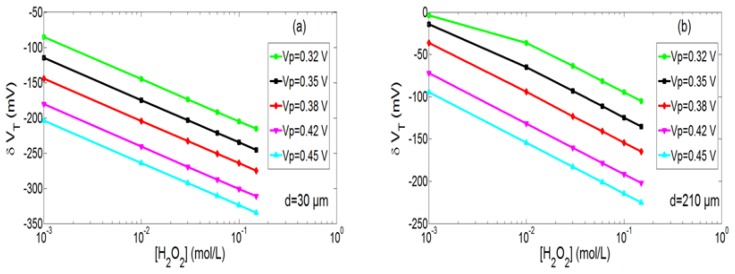
H_2_O_2_ analytical responses of the ElecFET microdevice (V_P_ = 0.32, 0.35, 0.38, 0.42, 0.45 V and t_P_ = 30 s) for two different distances between the integrated microelectrode and the pH-sensitive gate: (**a**) d = 30 μm and (**b**) d = 210 μm.

In order to quantify the pH variation, its minimal value and more precisely the associated minimal threshold voltage value have been studied according to [H_2_O_2_] concentration for two integration levels, *i.e.*, for two distances (d = 30 and 210 μm) between the integrated microelectrode and the pH-ChemFET gate sensitive area (Figure 10 respectively). Simulation results exhibit linear variations for several concentration decades (sensitivity ≈ 60 mV/decade). Such Nernstian sensitivity is related to linear variations of the [H_3_O^+^] concentration with the [H_2_O_2_] concentration at the microelectrode surface (*cf.* Equation (3)). This result demonstrates that the pH-ElecFET microdevice can be effectively used for the potentiometric detection of hydrogen peroxide. Furthermore, by increasing the polarization voltage V_P_ and/or the integration level, H_2_O_2_ detection range and detection limit can be improved.

## 4. Conclusions

Using MATLAB™ software, we have investigated the modelling of the ElecFET microdevice in the case of water electrolysis and hydrogen peroxide oxidation, taking into account chemical and electrochemical and acid/basic reactions in water phase as well as diffusion phenomena of water-based ions. In the first case, results show that the ElecFET phenomena depend strongly on the polarization parameters on the microelectrode (voltage V_P_ and time t_P_) as well as on dimensional characteristics related to the ElecFET functional integration (distance between the microelectrode and the pH-ChemFET gate sensitive gate area d). On one hand, according to the polarization sign, oxidation and reduction reactions of water are responsible for impulsional pH variations localized at the microscale (typical dimension: 1 mm and less). On the other hand, amplified phenomena are obtained when the polarization conditions and/or the integration level are increased.

In the second case, the ElecFET microdevice was shown to be compatible with hydrogen peroxide detection while using an indirect measurement technique based on the pH-related H_2_O_2_ oxidation products. Amplification phenomena were shown to have no influence on the detection sensitivity and Nernstian responses were finally evidenced for several concentration decades. Nevertheless, detection range variations and improved detection limits were still possible by increasing polarization conditions and/or integration level.

It should be mentioned that the ElecFET modelling was performed for solutions based on pure water and therefore without considering any buffer properties and/or any interfering pH variations. This choice was required to ElecFET detection/transduction principles, but it prevents us from comparing fully modelling and experimental results. As far as real samples are concerned, buffer properties will have to be considered to determine pH distributions in the ElecFET environment. Thus, the modelling will have to be adapted to each different buffer solution of interest. This can induce contradictory phenomena for the ElecFET behaviour. On one side, buffer properties can impede in some extents local pH changes, deteriorating detection properties in terms of sensitivity and/or detection range. On the other side, by impeding pH variations in solution, they can improve detection performances in terms of selectivity (especially to pH).

Nevertheless, the ElecFET microdevice is finally very promising in terms of applications, and modelling enables a complete comprehension of its detection principle based on a combination of amperometry and potentiometry. It allows a combination of the pH-ChemFET-metry technique and redox phenomena. Further studies will be focused on the modelling of the detection of H_2_O_2_-related biomolecules such as glucose as well as lactate, urate and/or glutamate ions by taking into account associated enzymatic reactions in buffered solution.
